# Modulation of microbiota, inflammation, iron status and gene expression of affected receptors in Parkinson’s disease rat model by synbiotic and dark chocolate

**DOI:** 10.1038/s41598-025-08740-6

**Published:** 2025-07-04

**Authors:** Sahar Y. Al-Okbi, Asmaa A. Ramadan, Rasha S. Mohamed, Hoda B Mabrok, Enas S. K. Al-Siedy

**Affiliations:** Nutrition and Food Sciences Department, National Research Centre, Cairo, Egypt

**Keywords:** Parkinson’s disease, Rats, Cecum microbiota, Inflammation, Oxidative stress, Iron status, Synbiotic, Dark chocolate, DMT1, DRD1, Biochemistry, Biomarkers, Medical research

## Abstract

Parkinson’s disease is among the neurodegenerative diseases that have no promising remedies. The present research is dealing with searching the associations between molecular, biochemical and microbiota changes in Parkinson’s disease rat model with and without intervention with dark chocolate as prebiotic or a mixture of probiotics (*Lactobacillus plantarum* EMCC 1039, *Lactobacillus acidophilus* and *Bifidobacterium lactis* BB12) during feeding a diet containing oat as prebiotic collectively designated as synbiotic (Sb). Four groups of rats were assigned; a normal control (C), a group given rotenone to induce Parkinson’s disease (P), and two test groups treated with rotenone; one received synbiotic (PSb) and the other treated by dark chocolate (PCh). Results showed plasma soluble transferrin receptor /log ferritin ratios that elevated in the P group denoting anemia was reduced in the test groups; with superiority to Sb. The increased brain malondialdehyde (MDA) together with the decreased glutathione (GSH) indicating high oxidative stress in the P group were improved in the test groups. Immune system that was affected in the P group by reduction of plasma CD4 which is the cluster of differentiation 4 and elevation of brain tumor necrosis factor-α (TNF-α) and plasma interferon γ (IFNγ) was improved in the test groups and completely amended concerning IFNγ; Sb showed more promising effect than chocolate concerning TNF-α and CD4. Up-regulation of the brain divalent metal transporter 1 gene (DMT1) in the P group was down-regulated significantly in the test groups till matching that of the C group. Down-regulation of brain dopamine receptor D1 (DRD1) gene expression was significantly up-regulated in the test groups with superiority of Sb. Brain histopathological changes in the P group were improved on treatment with either chocolate or Sb with more promising effect by Sb. The cecum content of Firmicutes (F) showed no difference among the different groups while Bacteroidetes (B) was significantly reduced on treatment with chocolate compared to all groups with significant increase of F/B. Cecum *Lactobacilli* showed significant increase in the PSb group compared to all other groups. It could be concluded that treatment with dark chocolate and Sb improved experimental PD with variable degrees.

## Introduction

Parkinson’s disease is a progressive neurodegenerative disease with α-synuclein aggregate deposition. Genetic and molecular pathology are among the main pathogenesis of PD with spreading of pathology between gut and brain. Maladaptive immune and inflammatory responses might be triggered in the gut and accelerate PD pathogenesis^1^. So far no specific therapy has been shown to be efficient for treating PD.

PD affects 1–2 per thousand of the world population. Substantia nigra with damage in the dopaminergic neurons of the CNS and lewybodies deposition are the main pathological alterations in PD as reviewed previously^2^. These changes were also accompanied by pathological alterations in the gastro intestinal tract with associated microbiota imbalance^2^. Therapies like catechol-O-methyltransferase inhibitors and anticholinergic drugs might increase the harmful flora, thereby increasing such unwanted microbiota imbalance and worsen the intestinal symptoms^2^. Treating PD patients with probiotic, prebiotic and synbiotics could reduce constipation, promote beneficial microflora and inhibit intestinal inflammation^2^. On the other hand gut dysbiosis was demonstrated to be linked to the occurrence and advancement of PD through inducing intestinal permeability, provoking neuro-inflammation, accumulating unusual levels of α-synuclein, reducing neurotransmitters’ creation and elevating reactive oxygen species^3^. The interpretation of PD pathogenesis based on the intestinal microbiome might lead to a novel prospective to explore new remedial approaches^3^ represented by probiotics as adjuvant therapy for improving constipation and reducing the progression of PD^4^.

PD has motor and non-motor symptoms. PD starts by the non-motor symptoms represented mainly by the intestinal changes then followed by the motor dysfunction that includes rigidity, tremors and bradykinesia^5^. Gut microbiota could influence the central nervous system via neurological and immune system pathways through microbiota-gut-brain axis. There is strong evidence that microbiota changes could mediate the incidence of PD, therefore influencing the microbiota changes by therapeutic agents could work as anti-PD^6^. In the present study it was decided to study the changes in immune system in the experimental PD through assessing CD4, brain TNF-α, plasma IFNγ and oxidative stress. The oxidative stress could be monitored by determination of brain malondialdehyde (MDA) and the reduced glutathione. It was also suggested that probiotics and prebiotic or combination of both as synbiotic could improve microbiota imbalance during PD in the current study.

Iron deposition represents critical pathological changes in PD patients^7^. In PD patients there is disturbance in iron distribution, transport, storage and circulation that certainly lead to increased iron deposition in the brain. Such iron accumulation could result in high oxidative stress, nerve cell death and mitochondrial dysfunction that contribute to PD pathology^7^. Therefore, the follow up of the degree of iron deposition during anti-PD novel therapeutic agent is a vital approach. Brain divalent metal transporter 1 (DMT1) gene is important to be studied in PD because it has a serious role in iron arbitrating neurodegeneration^8^. On the other hand, the interaction of immune system especially the inflammatory cytokines with iron status is an important association in PD^9^. On the other hand management of PD might be accomplished by up-regulation of gene expression of brain dopamine receptor D1 (DRD1)^10^. Therefore, the follow up of DRD1 is also necessary for studying the mechanism of action of novel anti-PD therapy.

Dark chocolate contains high percentage from cacao/ or cocoa, to which all the therapeutic effect of chocolate was ascribed. Cacao refers to cacao beans that have not been roasted while cocoa is made from bean that have been roasted. Dark chocolate as a prebiotic has been suggested to amend the pathological changes in PD^11^. Oat also is considered as an efficient prebiotic due to the presence of β-glucan that could increase the beneficial microbiota in the gut^12,13^.

The objective of the current work was to assess the therapeutic benefits of dark chocolate administration as prebiotic in comparison to synbiotic represented by *Lactobacillus plantarum*,* Lactobacillus acidophilus* and *Bifidobacterium lactis* as probiotic mixture with simultaneous administration of a diet containing oat as prebiotic in PD rat model. The therapeutic benefits were evaluated through assessing gut microbiota, iron status, immune alteration involving CD4 and brain inflammatory biomarkers represented by TNF-α and plasma IFNγ. DRD1 and DMT1 gene expression were also monitored during the study.

## Results

Phenolic contents and flavonoids were determined to be 5.32 ± 0.21 mg GA/g and 1.04 ± 0.02 mg QE/g, respectively in chocolate. Percentage DPPH scavenging activity of chocolate was 72.54 ± 0.88.

**Fig. 1:**
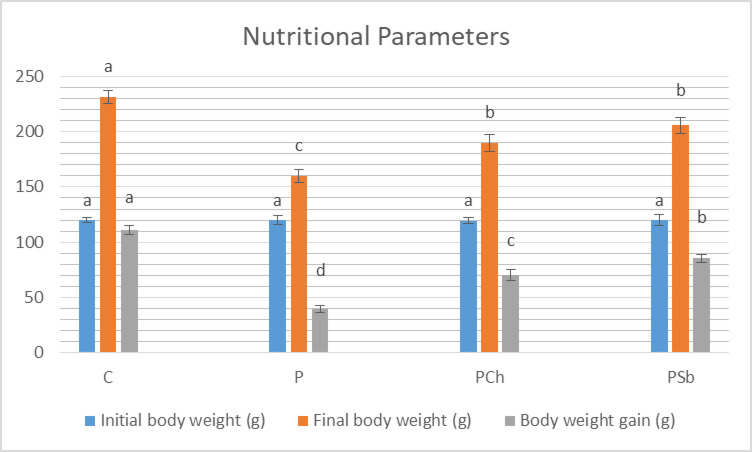
Nutritional parameters of different experimental groups. For each parameter, bars with different letters are significantly different at *p* ≤ 0.05. C: Normal control, P: Parkinson’s control, PCh: Parkinson’s rats with administration of chocolate, PSb: Parkinson’s rats with administration of synbiotic. SE is present above each bar (І).

It was noticed that both body weight gain and final body weight were significantly reduced in the P group when compared to the C group (Fig. 1). The PSb and PCh groups showed significant elevation in both parameters in comparison to the P group while exhibited significant reduction from the C group.

**Fig. 2:**
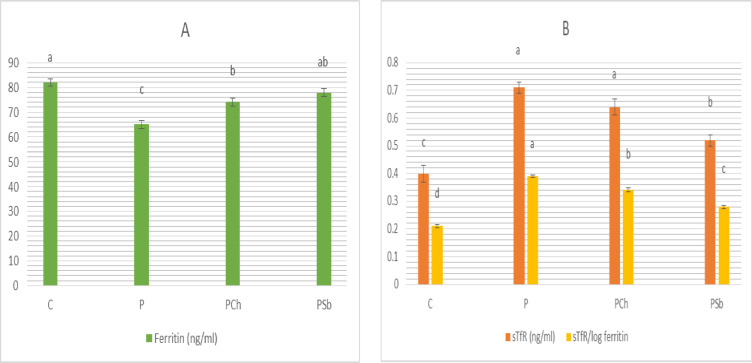
Iron status of different experimental groups. (**A**) Plasma ferritin; (**B**) Plasma sTfR and sTfR/log ferritin. For each parameter, bars with different letters are significantly different at *p* ≤ 0.05. C: Normal control, P: Parkinson’s control, PCh: Parkinson’s rats with administration of chocolate, PSb: Parkinson’s rats with administration of synbiotic. SE is present above each bar (І).

The results of iron status are clarified in Fig. 2. The P group showed significant decrease in plasma ferritin accompanied by significant elevation of soluble transferrin receptors (sTfR) and sTfR/log ferritin compared to the normal control group. The group treated by Sb showed significant decrease of soluble transferrin receptors compared to the Parkinsonism control while dark chocolate demonstrated insignificant decrease. The test groups demonstrated significant increases in ferritin in comparison to Parkinsonism control. The ratio sTfR/log ferritin was significantly decreased in the test groups in comparison to the P group; synbiotic was more efficient.

**Fig. 3:**
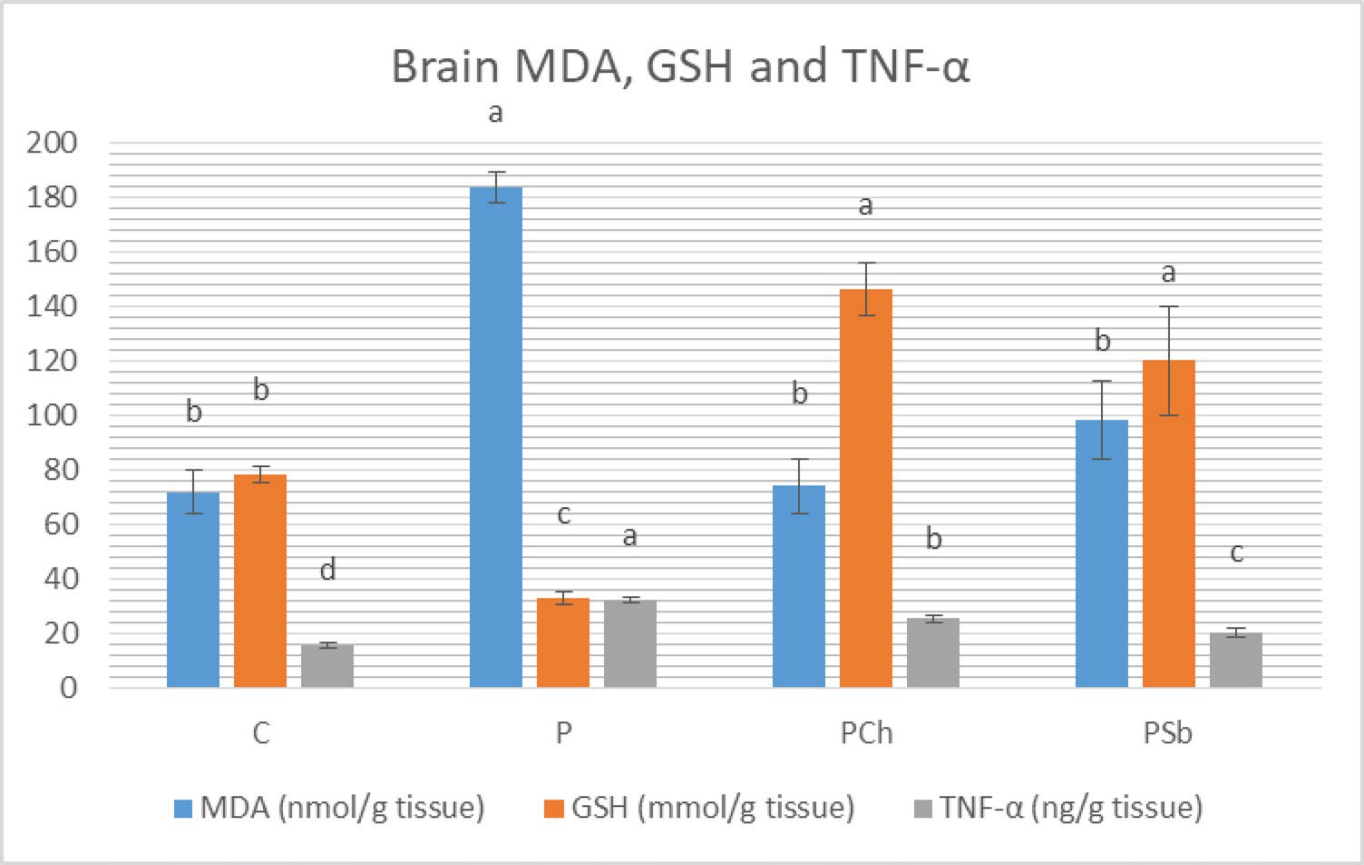
Brain malondialdehyde, reduced glutathione and TNF-α of different experimental groups. For each parameter, bars with different letters are significantly different at *p* ≤ 0.05. C: Normal control, P: Parkinson’s control, PCh: Parkinson’s rats with administration of chocolate, PSb: Parkinson’s rats with administration of synbiotic. SE is present above each bar (І).

It was noticed that brain MDA showed significant elevation associated with significant decrease in glutathione in the P group compared to the normal control group (Fig. 3). Both test groups exhibited significant decrease in MDA and elevation in glutathione when compared to the P group; MDA matched that in the C group while chocolate and Sb treated groups showed higher significant levels of GSH than the C group. Figure 3 demonstrated significant elevation in TNF-α in the P group compared to the normal control, while the two test groups demonstrated significant improvements of such parameters, with Sb to be more efficient than chocolate.

**Fig. 4:**
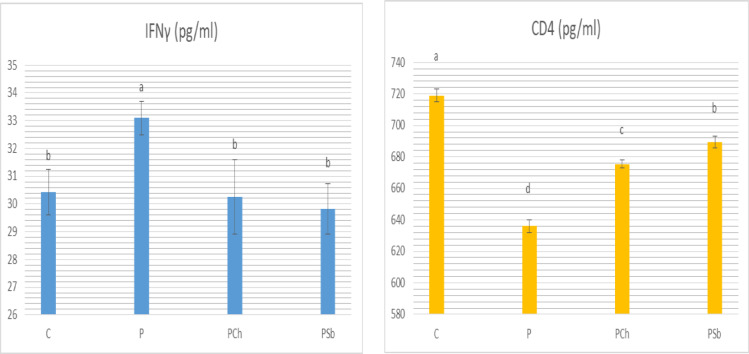
Plasma INFγ and CD4 of different experimental groups. For each parameter, bars with different letters are significantly different at *p* ≤ 0.05. C: Normal control, P: Parkinson’s control, PCh: Parkinson’s rats with administration of chocolate, PSb: Parkinson’s rats with administration of synbiotic. SE is present above each bar (І).

Plasma interferon-γ was increased significantly while CD4 was significantly reduced in the P group in comparison to the C group, meanwhile the test groups demonstrated significant improvement of both parameters (Fig. 4). Interferon-γ levels of the test groups were matching that of the normal control. A significant decrease in CD4 in the test groups was observed compared to the normal control group.

**Fig. 5:**
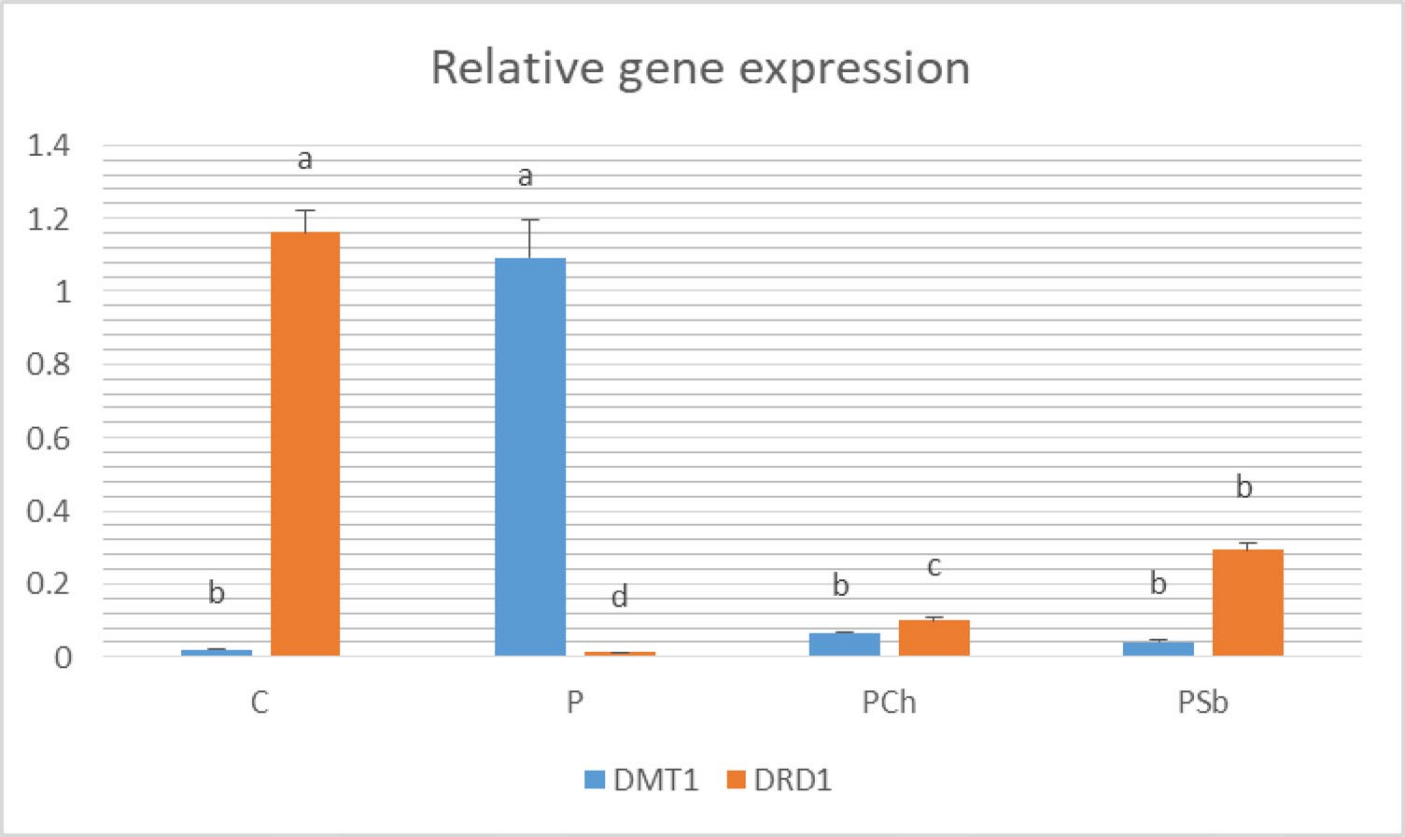
The relative expression of DMT1 and DRD1 genes in brain tissue of different experimental groups. For each parameter, bars with different letters are significantly different at *p* ≤ 0.05. C: Normal control, P: Parkinson’s control, PCh: Parkinson’s rats with administration of chocolate, PSb: Parkinson’s rats with administration of synbiotic. SE is present above each bar (І).

The gene expression of DMT1 (Fig. 5) was significantly up-regulated in the P group in comparison to the C group. Both Ch and Sb treatments induced significant down-regulation of the mRNA expression of DMT1 in comparison to the P group. The mRNA expression of The mRNA expression of DRD1 was down-regulated significantly in the P group compared to the C group. Significant up-regulation of DRD1 gene was noticed on treatment with either Ch or Sb compared to the P group.

Table 1 showed the microbiota in cecum content. It could be noticed that *Bacteriodetes* tended to decrease in the P group compared to the C group while significant decrease was noticed in the group treated with chocolate compared to the P group. The group given synbiotic showed insignificant change from the P group while demonstrated significant increase from rats treated with chocolate concerning *Bacteriodetes*. Insignificant changes were noticed in *Firmicutes* among the different groups. The only significant change concerning F/B ratio was related to the significant increase in the group given chocolate compared to all other experimental groups. The group treated with synbiotic showed significant increase in *Lactobacilli* compared to all other groups.

**Fig. 6:**
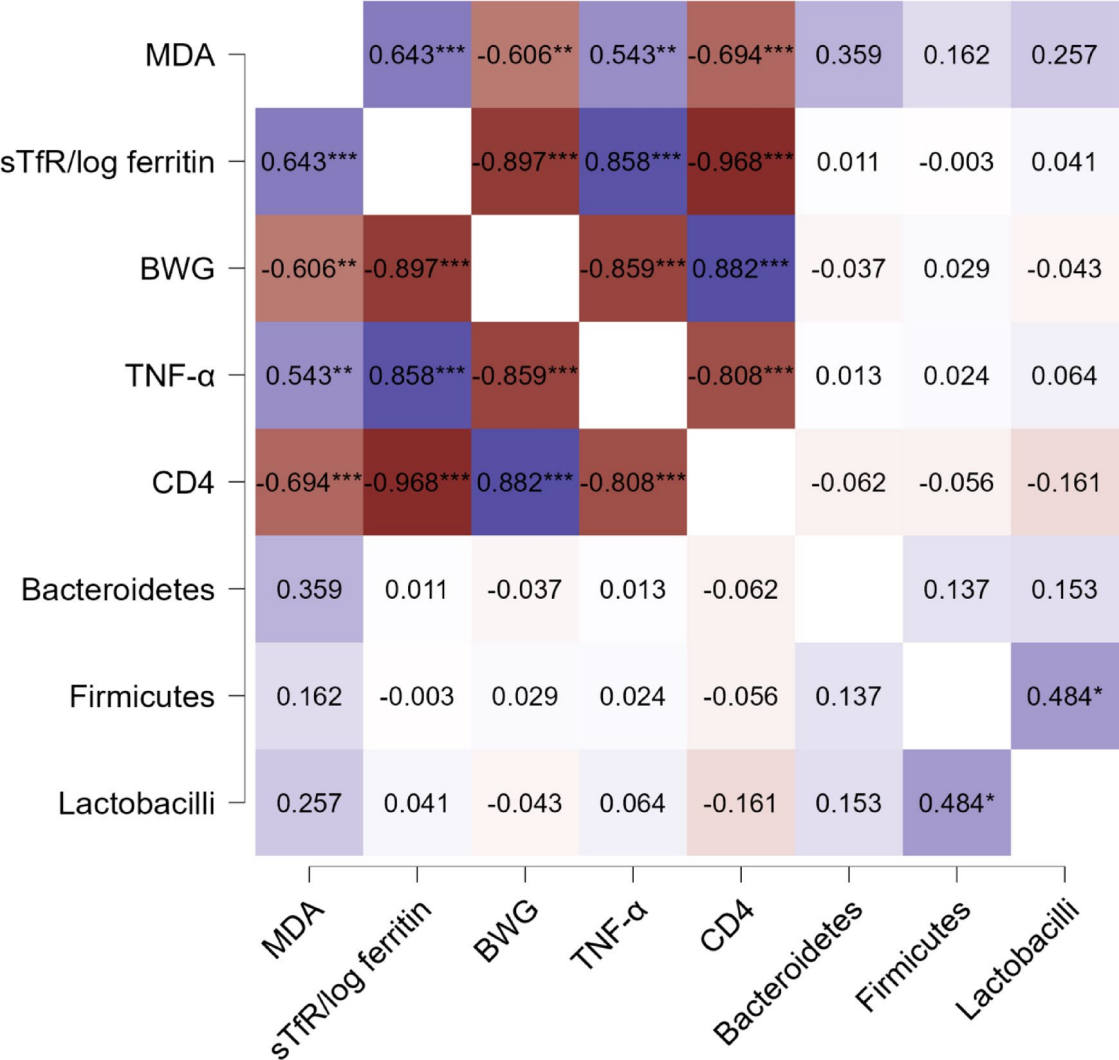
Spearman’s correlation coefficient (rho) Heatmap showing correlations between different parameters: Colors range from dark blue (strong positive correlation, i.e. *r* = 1.0) to dark red (strong negative correlation, i.e. *r* = − 1.0). The darker the colors the higher is the significance, *: *p* < 0.05, **: *p* < 0.01, ***: *p* < 0.001.

Correlation study (Fig. 6) clarified that MDA has significant negative correlation with BWG and CD4 and significant positive correlation with TNF-α and sTfR/log ferritin. The ratio sTfR/log ferritin demonstrated significant negative correlation with CD4 and BWG while exhibited significant positive correlation with TNF- α. BWG showed significant negative correlation with TNF- α with significant positive correlation with CD4.TNF-α demonstrated significant negative correlation with CD4. *Firmicutes* showed significant positive correlation with *Lactobacilli*.

**Fig. 7:**
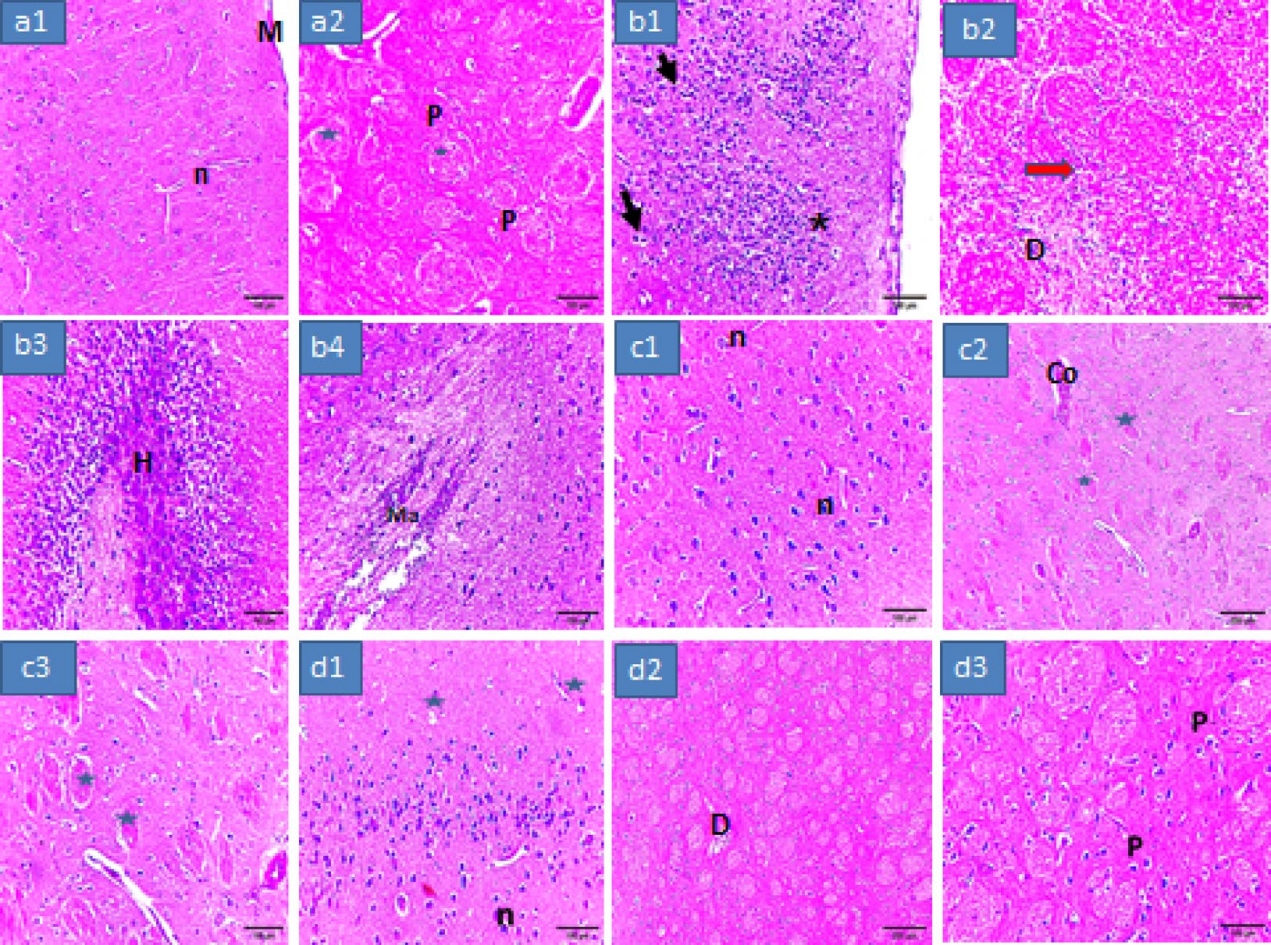
Brain histopathology of the different experimental groups. (**a1**) and (**a2**) Show normal appearance of the brain of the normal control group (X400) with noticeable intact neurons and cortex and normal nuclei (n), meninges (M), perikaryon (p) and myelination (star); (**b1**–**b4**) The brain sections of the P group (X400) showed severe changes, (**b1**) demonstrated neurons atrophy, degeneration and shrunken and bounded by microglial cells (arrow) with vacuolated neutrophil (asterisks) and accompanied by neurofibrillary tangles simulating lewy bodies, (**b2**) Neuroglial cells (red arrow) were severely accumulated with demyelination, (**b3**) The hippocampal region (H) displayed hemorrhage with blood vessel congestion in the striatum and midbrain, (**b4**) Cerebral malacia (Ma) is observed with tissue loss and gliosis accompanied with neuronal apoptosis; (**c1**–**c3**) Brain of rats received dark chocolate, (**c1**) (X400): The cerebral cortex showed decline in karyopyknotic neuronal cells, (**c2**) (X100), (**c3**) (X400): Displaying mild vacuolated neurtrophil and congestion (Co). (**d1**–**d3**) Rats treated by synbiotic, (**d1**) (X400): showing modest neuronal necrosis and degeneration with reduction of lesions meanwhile the cerebral cortex pyramidal cells appear normal. (**d2**) (X100), (**d3**) (X400) displaying reduced vacuolar spaces around perikaryon (P), and demyelination appears to be minimized.

Figure 7 demonstrated the brain histopathology of the different groups. The control rats with PD showed severe brain changes. Treatment with either dark chocolate or synbiotic produced improvement in the histopathological changes with superiority to Sb.

**Table 1:** Microbiota (*Bacteroidetes* and *Firmicutes* phyla and lactobacilli) in cecum contents (mean ± SE).

Microbiota				
Groups	Bacteroidetes (B) log cfu/g	Firmicutes (F) log cfu/g	F/B ratio	Lactobacilli log cfu/g
C	8.82^a^ ± 0.55	10.05^a^ ± 0.57	1.14^b^ ± 0.04	7.47^b^ ± 0.77
P	8.65^a^ ± 0.17	9.56^a^ ± 0.59	1.11^b^ ± 0.03	7.02^b^ ± 0.67
PCh	6.79^b^ ± 0.86	9.64^a^ ± 0.46	1.42^a^ ± 0.06	6.77^b^ ± 1.34
PSb	8.59^a^ ± 0.11	10.20^a^ ± 0.23	1.19^b^ ± 0.02	9.23^a^ ± 1.24

Different superscript letters in the same column are significantly different at *p* ≤ 0.05.

C: Normal control, P: Parkinson’s control, PCh: Parkinson’s rats with administration of chocolate, PSb: Parkinson’s rats with administration of synbiotic.

## Discussion

PD is considered a complex neurological aliment featured by loss of dopaminergic neurons in the substantia nigra with impairment of motor system function. Alteration of gut microbiota has been linked to PD which may open a new era towards therapeutic agents represented by probiotic, prebiotic and synbiotic that could influence gut-brain axis by affecting microbiota composition, enteric and central nervous system^14^. The gut microbiota is evolving as essential in modifying neurodegenerative diseases including PD via affecting the pathophysiology and the associated symptoms. PD symptoms often started by the gastrointestinal involving enteric nervous system. The dysbiosis might lead to pro-inflammatory condition that could be linked to gastrointestinal symptoms in PD patients^15^. Proinflammatory dysbiosis was reported to be present in PD patients and might trigger inflammation-induced misfolding of α-synuclein and development of PD pathology^16^. Alpha-synuclein is a 140 amino acid protein and a member of the synuclein family as reviewed by Butler et al.^17^. A hallmark of PD is a significant loss of dopaminergic neurons in the substantia nigra pars compacta, with some of the remaining neurons containing α-synuclein positive inclusions known as Lewy Bodies^18^ that could be observed in the brain histopathology of the PD control in the present study.

The most important bacterial phyla in gastrointestinal tract are Firmicutes (Bacillota) and Bacteriodetes. Firmicutes phylum includes two classes *Bacilli* and *Clostridia*. Under *Bacilli* presents orders like *Bacillales* (e.g. *Bacillus*, *Listeria* and *Staphylococcus*) and *Lactobacillales* (*Enterococcus*, *Lactobacillus*, *Luconostoc* and *Streptococcus*). Bacteriodetes phylum includes 4 main classes; *Bacteroidia* (*Bacteroides* spp.), *Cytophagia*, *Flavobacteria* and *Sphingobacteria*^19^. There are hundreds species under each phylum and the above mentioned are only the main classification. It is widely accepted that F/B has an important influence in maintaining normal intestinal homeostasis. Elevation and reduction of F/B ratio is considered dysbiosis, the reduction was observed in inflammatory bowel disease^20^. It was reported that there is an association between probiotic and F/B ratio via nutritional and immunomodulatory effect. However as the effects of different probiotics on the F/B ratio differ, selecting the appropriate species or mixture is essential. The most commonly tested probiotics for modifying the F/B ratio and treating intestinal inflammation are from the genus *Lactobacillus*^20^. Therefore, in the present research a mixture of different *Lactobacillus* species in addition to *Bifidobacterium lactis* were used as probiotic mixture in addition to oat as prebiotic collectively named synbiotic for ameliorating microbiota in PD; mean while dark chocolate was used as prebiotic in another group. However in the present study only insignificant reduction was noticed in F/B ratio in the P group compared to normal and even after treatment with synbiotic only insignificant increase was observed compared to the P group denoting the importance of assessing the different species in microbiota other than the whole phyla. Dark chocolate showed significant increase of F/B compared to the P group which might be better than Sb in this respect.

Prebiotic, represented by oat was demonstrated previously to improve microbiota balance by increasing the beneficial *Lactic acid* bacteria and *Bifidobacteria* due to its content from β-glucan^12,13^. In the current study *Lactobacilli* were significantly increased in the cecum content in the group treated by Sb compared to the P and even to the C group. Not only *lactobacilli* were increased but also the undetermined *bifidobacteria* might be increased because it is included in the given probiotic mixture. Synbiotic could directly and indirectly influence microbiota balance and immune response thereby confers health benefit to the host which might be reflected in improving IFNγ, TNF-α and CD4 and reducing oxidative stress (increasing brain GSH and reducing MDA) in the present study. The underlying mechanism is complex which might include an effect on colonic pH, inhibition of pathogenic bacteria, and production of antimicrobial molecules in addition to stimulation of beneficial bacteria growth. Immunological effect might also be related to improving gut barrier function, preventing pathogen translocation, modulating signal pathways, stimulating immune cell activities and balancing Th1/Th2 immune response^21^. Probiotics were shown previously to possess anti-inflammatory activity^22^ through increasing the release of the anti-inflammatory cytokine IL-10 via stimulation of CD4^23^ thereby reduce inflammation during PD. Specific compounds and numerous genes produced by probiotic could have immunoregulatory effect through modulation of systemic and mucosal immune cells and intestinal immune cells. Activation of toll-like receptors is among the immune regulation of probiotic and prebiotic^24^. Although dark chocolate increased F/B ratio, however it did not produce any significant change in *Lactobacilli* compared to the P group. Rats with induced PD that were given the synbiotic demonstrated significant increase in *Lactobacilli* compared to those treated by dark chocolate. The significant positive correlation between *Lactobacilli* and Firmicutes observed in the present study could be explained on the basis that *Lactobacilli* are species included within such phyla.

It was reported that probiotic specially *Lactobacilli* and *Bifidobacteria* have antioxidant effect by producing metabolites and antioxidant enzymes, enhancing antioxidant capacity and reducing host oxidant metabolites^25^. Probiotic supplementation significantly improved inflammatory biomarkers like TNF-α^26^. The term psychobiotics refers to probiotics, prebiotics and other treatments that manipulate the signals between the gut and brain mediated by microbiota to improve mood, cognition and anxiety. The psychotherapeutic effect is reflected in stimulation of neurotransmitters, short chain fatty acids, anti-inflammatory cytokines and neurohormones. The metabolites produced by psychobiotics can cross the blood brain barriers and modulate the immune system via communicating the nervous system through gut-brain-axis. Psychobiotics also has the ability to reduce brain inflammation and oxidative stress. The most studied psychobiotics belongs to genus *Lactobacillus* and *Bifidobacterium*. The probiotics used in the present study *Lactobacillus plantarum*,* Lactobacillus acidophilus* and *Bifidobacterium lactis* are among the main reported psychobiotics as reviewed by Dhyani et al.^27^ that could be used for treatment of neurodegenerative diseases including PD.

Undernourishment as in case of PD that could be reflected in reduced body weight in the P group in the present study might be attributed to intestinal high oxidative stress and inflammation that lead to reduction of nutrients’ absorption. This is supported by the significant negative correlation between body weight gain and both MDA and TNF-α observed in the current study. Administration of synbiotics could elevate the intestinal and systemic immune responses and repair the damaged histology of intestine and thymus^23^ thereby improved malnutrition represented by the increased body weight as seen from the present study. This explanation could also be applied on the group treated by chocolate as prebiotic that showed significant increase in body weight. A mixture of *L. acidophilus* and *Bifidobacterium infantis* was shown previously to improve gastrointestinal non-motor symptoms in PD patients^28^. This may participate in improving body weight in the present study due to presence of *L. acidophilus* in the probiotic mixture. Improvement of constipation in PD patient was reported during administration of fermented milk containing mixture of probiotic and prebiotic fiber^29^ which subsequently improved appetite and body weight. The ratio sTfR/log ferritin demonstrated significant negative correlation with body weight gain in the current study which showed that the presence of anemia could have a hand in reduced body weight which could be attributed to reduction of iron status.

In a previous research *L. plantarum CCFM405* improved the rotenone-induced motor deficits and constipation, reduced dopaminergic neuronal death, decreased intestinal inflammation and neuroinflammation and elevated dopamine levels, 5HT and accompanying metabolites in the brain of mice with PD. *L. plantarum CCFM405* was reported to normalize the gut microbiota through increasing the relative abundance of *Bifidibacterium*, *Turicibacter* and *Faecalibaculum* and reducing relative abundance of *Alistipes*, *Bilophila*, *Akkermansia* and *Escherichia-Shegilla*. The authors suggested that *L. plantarum CCFM405* prevent experimental PD by modulating gut microbiota-metabolite axis and by increased branched chain amino acids biosynthesis with dominant role in neuroprotection^30^. The probiotic used in the present study contains *L. plantarum* that could elicit its therapeutic effect towards PD by similar mechanism.

The elevated sTfR and sTfR/log ferritin in the P group have been previously reported as good biomarker of iron deficiency anemia even during inflammatory condition^31^.The anemia notified by reduction of plasma ferritin and the increase of sTfR and sTfR/log ferritin in PD model in the present research might occur due to disturbance in iron distribution, transport, storage and circulation in PD as reported previously^7^. Increased iron deposition in the brain of PD patients as reported by Zeng et al.^7^ could share in the development of anemia and the pathology of the disease via elevating brain oxidative stress and neuronal death as could be seen by elevated MDA, reduced GSH and the brain histopathology in the PD model in the current study. The significant positive correlation between MDA and sTfR/log ferritin as seen in the present research might support the elevated oxidative stress with the presence of anemia. Expression of brain DMT1 gene might have an important role in inducing iron-mediating neurodegeneration^8^. The elevated DMT1 in PD model in the current research with the simultaneous anemia could support the aforementioned assumptions. It is worth mentioning that the elevated TNF-α could stimulate the interaction of hepcidine (iron regulatory hormone) with ferroportin (the iron exporter) causing iron sequestration. Therefore anemia could result from impaired iron hemostasis and the inhibiting effect of pro-inflammatory cytokines on erythropoiesis in addition to alterations in the erythrocyte membrane that impairs its survival^9^. The significant positive correlation between TNF-α and sTfR/log ferritin observed in the present study confirms the association between inflammatory cytokines (as part of immune system) and iron status. Based on this explanation the improved iron status together with the down-regulation of DMT1 and the reduction of TNF-α, INFγ and the oxidative stress on administration of either Sb or dark chocolate demonstrated such associations with improvement of PD pathology which was mirrored in the amendment of the brain histopathological changes. Short chain fatty acids producing probiotics like *Lactobacillus plantarum* enhances iron absorption. Also, *Lactobacillus acidophilus* and *Bifidobacterium longum* improve iron absorption and control the course of anemia. Probiotic metabolites (postbiotics) including vitamins, short chain fatty acids and tryptophan are involved in the regulation of iron intestinal absorption and may influence iron status^32^, this may have a hand in the improved iron status on administration of Sb.

Brain dopamine receptor D1 modulates excitatory effects on neurons^33^. DRD1 agonist has remedial effect towards PD, produce cognitive enhancement and neuroprotective effect by suppressing reactive oxygen species and exerting anti-apoptotic activity. The significant positive correlation between MDA and inflammatory cytokine (TNF-α) showed in the present study reflected the association between oxidative stress and inflammation. Reactive oxygen species represented by hydrogen peroxide can induce inflammation through activation of transcription factor NF-*k*B^34^. Therefore DRD1 agonist that is capable of reducing oxidative stress could subsequently have anti-inflammatory activity. The up-regulation of DRD1 gene on administration of either Sb or dark chocolate might speculate that both can work as DRD1 agonists.

CD4 is a glycoprotein that serves as a co-receptor for the T-cell receptor. CD4 which is a type of T-cell that plays an important role in the adaptive immune system showed alterations in PD patients. Immune dysregulation has been suggested to play a role in the progressive degenerating aspects of PD with the hypothesis that interaction between microgalia and infiltrating T-cells shape the pathogenesis of PD^35^. In the present study, inverse significant correlations are noticed between CD4 with MDA, TNF- α and sTfR/log ferritin denoting that oxidative stress, inflammation and anemia in PD are associated with reduced CD4. On the other hand, body weight gain showed significant positive correlation with CD4. It is reported that therapies that modulate CD4 were suggested to produce neuroprotection in PD patients^35^. Therefore Sb and chocolate administration that produced an increase in CD4 are considered efficient therapy for PD.

PD produces a gradual loss of nerve cells and drop in dopamine level. Different components that present in cocoa (present in dark chocolate) can cross the blood-brain barrier and can improve mood and cognitive function such as theobromine, caffeine and phenylethylamine reported to enhance dopamine release. Phenylethylamine is not only a precursor of dopamine, but it also enhances the release of norepinephrine and dopamine into synaptic cleft^36^ leading to feeling of well-being. A biogenic amine in dark chocolate can substitute dopamine and improve PD symptoms^37^. Theobromine is an adenosine receptor antagonist like caffeine and possesses in addition antioxidant and gene expression regulation properties^38^. Also, it induces the expression of brain derived neurotrophic factor which is linked to the improved motor learning in PD mouse model^39^. Chocolate contains tyrosine as a dopamine precursor, serotonin and endorphins that contribute to appetite and mood regulation^40^. Clovamide (N-caffeoyl-L-DOPA), a conjugate of L-DOPA extracted from cocoa beans has an in-vitro neuroprotective activity^41^. Methylxanthines a potential flavonoid present in dark chocolate is considered as dopamine precursor and the controlled intake of dark chocolates can replace L-dopa^11^. Dark chocolate is rich in phenolic compounds and flavonoids as shown from the present study with antioxidant and anti-inflammatory activity therefore improves nerve degeneration and blood supply to the nerves. The antioxidant activity was confirmed in both the in-vitro DPPH scavenging activity and the in-vivo study in the current research.

## Conclusion

The determined cecum microbiota represented by Firmicutes, Bacteriodetes, F/B and *Lactobacilli* showed insignificant changes in the PD rat model compared to the normal control however it did not prove or negate changes of other undetermined species. The expression of brain DMT1 gene was increased with concomitant reduction of DRD1 in PD model. Iron status symbolized by sTfR/log ferritin reflects the occurrence of anemia in PD model. The elevated brain TNF-α and plasma IFNγ with simultaneous reduction of plasma CD4 demonstrated inflammation with alteration in immune system specially with the increased oxidative stress denoted by brain MDA elevation and GSH reduction. Severe brain histopathological changes were noticed in PD model. Administration of either dark chocolate or synbiotic produced significant improvements in the changes occurred during PD. Sb showed superiority in reducing anemia and TNF-α and elevating CD4 and DRD1 gene expression and improving brain histopathological alteration compared to dark chocolate. Cecum *Lactobacilli* showed significant increase in the PSb group compared to all other groups. Bacteroidetes was significantly reduced on treatment with dark chocolate compared to all groups with significant increase in F/B. The probiotic mixture and prebiotics used in the current research could be utilized as a potential food supplement in PD management. Future researches should focus on the exploration of specific gut microbiota and their metabolites rather than the whole phyla to recognize which exactly could contribute to the development of PD. It would be appropriate also to quantify the M1 macrophage markers in the microglia and the related inflammatory cytokines in addition to quantification of M2 macrophage and the associated anti-inflammatory cytokines with the evaluation of M1/M2 polarization in a prospective study.

## Materials and methods

### Materials

- Rotenone was purchased from Abcam, USA for induction of PD. All chemicals used in the study were of high grade and purity.
- Probiotic strains: *Lactobacillus acidophilus* (Chr. Hansen’s Lab., Denmark), *Lactobacillus plantarum* EMCC 1039 and *Bifidobacterium lactis* BB12 were kindly supplemented from the Dairy Science Department, National Research Centre, Egypt.
- Brown chocolate J.D Gross, ARRIBA: Dark chocolate containing 95% Cacao was obtained from Lidl U.K. GmbH and Lidl Britain Ltd. Wimbledon, London.
- Skimmed milk was obtained from local market, Egypt.

### Methods

#### Total phenolics, total flavonoids, and free radical scavenging activity of chocolate

Dark chocolate (0.1 g) was extracted with 2.0 mL of 80% aqueous ethanol overnight on a platform shaker. The mixture was centrifuged, and the residue was re-extracted in an ultrasonic water bath for 5 min. The mixture was centrifuged and the supernatants obtained from each run were combined.

Total phenolics were determined colorimetrically in the dark chocolate according to the modified Folin-Ciocalteu micro method^42^. Absorbance was measured at 760 nm using a UVPC spectrophotometer. Gallic acid (GA) was used as a standard for the calibration curve. Total phenolic content was expressed as mg GA equivalent/g dry chocolate. Total flavonoids were determined in the dry chocolate^43^ with the use of quercetin as a standard for the calibration curve. Total flavonoids content was expressed as mg quercetin equivalent (QE)/g dry chocolate. DPPH free radical scavenging activity of chocolate was measured according to a previously described method^44^ with some modifications; the absorbance was recorded at 515 nm. Blank was run in parallel in an identical manner without test samples. The percentage of DPPH radical-scavenging activity was calculated as follows: [(A control – A sample)/A control] × 100, where A control was the absorbance of the control reaction (containing all reagents except the test compound), and A sample is the absorbance of the test sample.

#### Preparation of rotenone for induction of parkinsonism in rats

Rotenone was prepared using dimethylsulfoxide (DMSO) and olive oil according to Mbiydzenyuy et al.^45^. The vehicle of the previously prepared solution was prepared without rotenone to be used for the control group.

#### Preparation of probiotic mixture and dark chocolate for dosing rats

- *Lactobacillus plantarum* EMCC 1039, *Lactobacillus acidophilus*,* Bifidobacterium lactis* BB12 cultures were freshly prepared by inoculating 1 ml from the stock of each strain into 100 ml of sterile skimmed milk and incubated at 37 °C for 48 h under anaerobic conditions to obtain a final concentration of 1 × 10^8^ cfu/ml from each strain. One ml of each strain culture was mixed immediately before giving the mixture orally to the rats so that each rat got 3 ml of the mixture daily.
- Dark chocolate was grinded to powder and dissolved in warm water to be given to rat as 500 mg/kg rat body weight.

#### Preparation of diet

Two types of diets were prepared, a balanced diet^46^ and a balanced diet containing 10% oat as a prebiotic (Table 2).

**Table 2:** Diet composition (g/100 g).

Ingredients	Balanced Diet	Oat containing diet
Casein	12	12
Sunflower oil	10	10
Starch	68.5	58.5
Cellulose	5	5
Salt mixture	3.5	3.5
Vitamin mixture	1	1
Oat	-	10

#### Animal experiment

Male adult Wistar rats of body weight ranging from 115 to 130 g (8–9 weeks of age). were purchased from Animal House of National Research Centre, Egypt. Handling and care of animals were carried out according to the Medical Research Ethics Committee, National Research Centre, Cairo, Egypt (Approval No. 19/175), and followed the recommendations of the National Institutes of Health Guide for Care and Use of Laboratory Animals (Publication No. 85 − 23, revised 1985). The study is reported in accordance with ARRIVE guidelines. Four groups of rats were assigned, each group comprised 8 rats. The first group was designated as the normal control (C), the second group served as the Parkinson’s control group (P). The third and fourth groups represented the test groups. Rats of the first, second and third groups were maintained on a balanced diet al.l over the experiment (4 weeks) while rats of the fourth group were fed on a balanced diet containing 10% oat. Rats of the third group (PCh) were treated orally from the first day of the experiment with dark chocolate as 500 mg/kg on a daily basis till the end of the experiment. Probiotic mixture was given as daily oral dose (as 1 × 10^8^ from each strain) to rats of the fourth group (PSb) all over the whole experiment. At the start of the fourth week, rats of groups 2, 3 and 4 were treated subcutaneously with rotenone as 2.5 mg/kg rat body weight/day for 7 consecutive days for induction of PD. Rats of the C group were injected subcutaneously by the same quantity of the vehicle used for the preparation of rotenone on a daily basis in the last week of the experiment. Rats were weighed once a week. At the end of the experiment, body weight gain (BWG) for each rat was calculated. All rats were fasted for 16 h. The fasted rats were anesthetized by pentobarbital and blood samples were collected in heparinized tubes and centrifuged at 3000 rpm for 15 min to obtain the plasma. Plasma soluble transferrin receptor (sTfR), ferritin, IFN-γ, and CD4 were assessed using ELISA kits supplied from Sunlog Biotech Co. LTD, China. Euthanasia was carried out by cervical dislocation under anesthesia. The brains of all rats were excised and placed immediately on ice, and cut into two longitudinal sections. One section was used for the assessment of malondialdehyde (MDA)^47^, reduced glutathione (GSH)^48^, and TNF-α, adopting the method of ELISA kit purchased from Sunlog Biotech Co. LTD, China. Gene expressions of brain divalent metal transporter 1 (DMT1) and dopamine receptor D1 (DRD1) were assessed using real time polymerase chain reaction (RT-PCR). The other section of the brain was fixed in 10% formalin for histopathological examination^49^ using hematoxylin and eosin. Cecum contents were obtained for investigation of microbiota represented by *Firmicutes*,* Bacteriodetes and Lactobacilli* using RT-PCR.

### Assessment of DMT1 and DRD1 gene expression by RT-PCR

PureLink^®^ RNA Mini Kit (Ambion^®^ Life TechnologiesTM) was used to separate total RNA from brain tissue, according to the manufacturer’s instructions. Exactly 1.5 µg from total RNA was used to synthesize cDNA using RevertAid first strand cDNA synthesis kit (Thermo Fisher^®^ InvitrogenTM). RT-PCR was carried out using RotorGene^®^ MDx instrument. The reaction mixture of the RT-PCR (25 µl) contained 1 µl template cDNA, 1× the EvaGreen^®^ PCR master mix (HOT FIREPol^®^ EvaGreen^®^ qPCR Mix Plus, Solis BioDyneTM) and 0.2 µM of the primer pairs. Table 3 shows the primer pairs sequences applied for DMT1 and DRD1. PCR reactions were implemented using the following condition: 50 °C for 2 min, 95 °C for 12 min, 45 cycles of 15 s at 95 °C, 30 s at 60 °C, 30 s at 72 °C, melting curve program (60–95 °C). For negative control PCR water was used instead of cDNA templates. The target gene relative expression was calculated using 2-∆∆CT method^50^. The expression of the target gene was normalized to the expression of the house-keeping gene GAPDH.

**Table 3:** The primers used for amplifications in RT-PCR for brain DMT1 and DRD1.

Target genes	Sequences	Product size (bp)
DMT1	FW (5′-TTTGGCTTTCTCATCACTATCATGGC − 3′) RW(5′-ATTGGCTTCTCGAACTTCCTGCTTATTGGC-3′)	248
DRD1	FW (5′-TCCTTCAAGAGGGAGACGAA-3′) RW(5′- CCACACAAACACATCGAAGG-3′)	168
GAPDH	FW (5′- GTATCGGACGCCTGGTTACC-3′) RW(5′- CGCTCCTGGAAGATGGTGATGG-3′)	202

DMT1: Divalent metal transporter 1, DRD1: Dopamine receptors D1, GAPDH: Glyceraldehyde-3-phosphate dehydrogenase, bp: base pair.

### Real time PCR analysis of microbiota in cecum content

Total genomic DNA was extracted from rat cecum content samples according to the manufacturer’s protocol (i-genomic stool DNA extraction mini kit, iNtRON biotechnology, South Korea). The DNA concentration and purity (A260/A280 ratio) were measured by NanoDrop spectrophotometer. DNA samples were analyzed for quantitative determination of Firmicutes and Bacteroidetes phyla and *Lactobacillus* genus using real time PCR. Real-time PCR was implemented using the Applied Biosystems QuantStudio™ 5 Dx System (Thermo Fisher Scientific, USA). The reaction was performed in a total volume of 20 µL containing 2 µL genomic DNA, 4 µL EvaGreen^®^ qPCR master mix plus (Solis BioDyne, Estonia) and 0.5 µL of each of the forward and reverse primers. Specific oligonucleotide primers were adopted from previous studies (Table 4). Primer sequences were confirmed for specificity using NCBI BLAST database. The PCR reaction conditions for the amplification of DNA were 50 °C for 2 min, 95 °C for 15 min, 50 cycles each of 15 s at 95 °C, 60 s at 60 °C, 15 s at 72 °C, melting curve program 55–95 °C. Genomic DNA was extracted from an activated culture of *Lactobacillus acidophilus* B-4242 and was used as standard. *Lactobacillus* specific primer sequence was implemented from a previous study (Table 4). Bacterial genomic DNA from *L. acidophilus* was used to prepare 10-fold dilution series copies to generate a standard curve of cycle threshold values (Ct) against bacterial copy number. Sample copy numbers were obtained from the standard curve then normalized to gram of cecum content. All primer sequences were confirmed for specificity using NCBI BLAST database. The calculations were done using the Applied Biosystems software (Design & Analysis v2.6.0).

**Table 4:** Oligonucleotide sequence of primers used in the RT-PCR analysis.

Primer name	Primer sequence 5′–3′	Annealing temperature (°C)	Amplicon size (bp)	References
Bacteroidetes F	GTTTAATTCGATGATACGCGAG	58	122	^51^
Bacteroidetes R	TTAASCCGACACCTCACGG			
Firmicutes F	GGAGYATGTGGTTTAATTCGAAGCA	57	126	^52^
Firmicutes R	AGCTGACGACAACCATGCAC			
*Lactobacillus* F	GGAATCTTCCACAATGGACG	60	217	^53^
*Lactobacillus* R	CGCTTTACGCCCAATAAATCCGG			

F: Forward primer, R: Reverse primer, bp: base pair.

### Statistical analysis

The data were expressed as mean ± standard error of the mean (SE) and analyzed with one-way analysis of variance (ANOVA) followed by the Tukey’s post-hoc test. Differences were considered significant at *P* ≤ 0.05. Whenever the data was not normally distributed, non-parametric Kruskal-Wallis’s test was used to compare the means followed by Mann Whitney’s test for individual comparisons. Spearman’s rank test was used to detect correlations between different parameters. Data analysis was done using JASP software Version 0.19.

## Data Availability

All data generated or analyzed during this study are included in this published article.
